# Remote Cameras Reveal Experimental Artifact in a Study of Seed Predation in a Semi-Arid Shrubland

**DOI:** 10.1371/journal.pone.0165024

**Published:** 2016-10-20

**Authors:** Alissa J. Brown, Douglas H. Deutschman, Jessica Braswell, Dana McLaughlin

**Affiliations:** 1 Department of Biology, San Diego State University, San Diego, California, United States of America; 2 Environmental Science Department, Queens University of Charlotte, Charlotte, North Carolina, United States of America; 3 SoCal Biological Consulting, Chula Vista, California, United States of America; University of Missouri Kansas City, UNITED STATES

## Abstract

Granivorous animals may prefer to predate or cache seed of certain plant species over others. Multiple studies have documented preference for larger, non-native seed by granivores. To accomplish this, researchers have traditionally used indirect inference by relating patterns of seed removal to the species composition of the granivorous animal community. To measure seed removal, researchers present seed to granivorous animals in the field using equipment intended to exclude certain animal taxa while permitting access to others. This approach allows researchers to differentiate patterns of seed removal among various taxa (e.g., birds, small mammals, and insects); however, it is unclear whether the animals of interest are freely using the exclusion devices, which may be a hindrance to discovering the seed dishes. We used video observation to perform a study of seed predation using a custom-built, infrared digital camera and recording system. We presented native and non-native seed mixtures in partitioned Petri dishes both within and outside of exclusion cages. The exclusion cages were intended to allow entrance by rodent taxa while preventing entrance by rabbits and birds. We documented all seed removal visits by granivorous animals, which we identified to the genus level. Genera exhibited varying seed removal patterns based on seed type (native vs. non-native) and dish type (open vs. enclosed). We documented avoidance of the enclosed dishes by all but one rodent taxa, even though these dishes were intended to be used freely by rodents. This suggests that preference for non-native seed occurs differentially among granivorous animals in this system; however, interpretation of these nuanced results would be difficult without the benefit of video observation. When feasible, video observation should accompany studies using *in situ* equipment to ensure incorrect assumptions do not lead to inappropriate interpretation of results.

## Introduction

The composition and structure of plant communities are subject to a variety of top-down (e.g., herbivory, seed predation) and bottom-up (e.g., resource availability) effects [1,2]. The influence of seed predators on plant communities is particularly important but challenging to address, given the difficulty in tracking seed fates and understanding behaviors of granivorous animals, which are often elusive, nocturnal, or rare. In one dramatic study, 12 years of excluding kangaroo rats from semi-arid shrubland plots resulted in a complete shift in plant community composition to that of an annual grassland [3]. These results imply that plant species whose seeds are subject to intense, selective granivory may be inhibited, even if they are otherwise good competitors. Selectivity in seed choice–among other behaviors–by granivorous animals can therefore influence competitive dynamics in plant communities, provide more opportunities of co-existence for less competitive species, and influence the extent of invasion of non-native plants [4–7]. As a result, studies of selective seed predation that aim to document these effects are numerous in ecological literature, and the methods used to address these challenging questions are varied (e.g., [8–11]).

To study seed predation, researchers often use exclosure cages manipulated in various ways to allow access to certain animal taxa, thus allowing them to parse out relative seed removal among multiple taxa. For example, Kelt et al. [12, 13] and Braswell [14] use PVC tubes with a bend at a 90-degree angle as the only access point to wire mesh cages containing a seed dish. This design prevents access to the enclosed seed dish by birds and rabbits while permitting access to rodents. The granivorous rodents are therefore attributed as the guild responsible for any seed removal from the enclosed seed dish. The premise of this notion is conditional upon the following assumptions: 1) the equipment is not allowing access to birds and rabbits; and 2) the equipment is not inhibiting or discouraging access to rodents.

To validate the assumption that taxa are using the experimental equipment as intended, researchers will often pilot test the seed stations, thereby directly observing their use by the taxa of interest. These observations can confirm that the taxa of interest are capable of using the equipment and that the exclosure treatment is excluding unwanted taxa (i.e., assumption 1). However, granivorous animals may not be using the exclosure cages freely (assumption 2), and this behavioral nuance is more difficult to observe.

If exclosure treatments inhibit use by the species of interest, researchers may underestimate–or otherwise incorrectly quantify–the amount of seed removed by the target community. For example, by excluding birds and rabbits from caged exclosures, the target community (rodents) may avoid using the exclosures and favor removing seed from dishes open to all taxa. While researchers may interpret seed removal from the caged seed dishes as removal by the entire rodent community, this removal may actually be from a subset of the rodent community. Without video observation of seed removal, it would be difficult to determine whether seed removal from the caged dish represents that of a subset of rodents proportional to those present in the study site, or a subset not representative of the granivorous rodent community. If the latter occurs, patterns of seed removal would be influenced by experimental artifact.

Exclosure treatments intending to parse out relative contributions of seed removal patterns by granivorous taxa primarily focus on separating removal based on coarse taxonomic units (small mammals, birds, and ants) ([15–17]; but see [13]). Although different species or genera may be more important players in seed removal than others, this notion would necessarily be removed from consideration using standard exclosure techniques. Using more complex exclosures, researchers can tease apart seed removal between rodent genera of different sizes [13]; however, these studies still depend on assumption 2 (i.e., that genera are freely using exclosures intended for them).

We deployed seed predation stations with two types of seed dishes: one open to all granivorous animals; the other intended to exclude all but rodents and insects. We recorded all visitations to the seed predation stations using a custom-built, infrared digital camera and digital recording system. Video observation allowed us to couple mass of seed removed from dishes with video observation of seed removal patterns of the granivorous community. To our knowledge, this study is the first to test the assumption that rodents freely use exclusion equipment in a study of seed predation. Furthermore, identification of granivorous animals to the genus level allowed us to observe patterns of seed removal at smaller taxonomic units than is typical in studies of seed predation.

## Materials and Methods

### Study Area

The study site is located at Rancho Jamul Ecological Reserve (RJER) in eastern San Diego County, California (32° 41 ‘N, 116°52’ W, 300 m elevation) and is managed by the California Department of Fish and Wildlife, which permitted this work. The site was designated as an Ecological Reserve in August 2000 and previously used for farming and grazing [18]. Although the site was historically chaparral and coastal sage scrub, non-native plant species have invaded much of the reserve, particularly invasive grasses from the genera *Bromus* and *Avena*, and forbs such as *Erodium* and *Brassica*. Frequent wildfires in the area have contributed to this invasion: The 2003 Cedar and Otay wildfires burned eighty percent of the land, and the 2007 Harris fire burned about half the plots [19, 20].

### Seed Predation

For a previous study, sixteen 49 x 49 meter grids were established at RJER before 2003 to monitor responses of the small mammal and plant communities to non-native plant invasion [14]. We were able to locate 14 of the original grids and use most of the original equipment for the current study. Across the approximately 2,266-hectare reserve, the grids were established in areas of varying slope, aspect, plant community composition, and topography representative of many semi-arid shrublands of southern California. The grids were not located closer than 150 meters from one another, with a typical distance of 500 meters apart.

We deployed two seed stations per grid, typically located on opposite corners of each grid (about 69 m apart). Each seed station consisted of two seed dishes placed approximately 40 cm apart; one open to all animals, and one enclosed in a wire mesh cage. The exclosure cages were constructed from hardware cloth (1cm mesh), with dimensions of about 30cm in height and 15cm in diameter. A hole was cut into the side of the cages, where the polyvinyl chloride (PVC) tube (10cm in diameter and 60cm in length) was inserted as the only entrance for non-insect animals. Each PVC tube has a 90-degree bend in the middle to exclude birds and rabbits [12–14].

We presented seed in partitioned Petri dishes, each with a mixture of native seed on one side and a mixture of non-native seed on the other. To prevent spilling, we drilled a hole through the center of a Petri dish lid, fixed the lid (lips facing upward) into the ground with a nail, and set the seed dish on the lid. Seed mixtures were composed of species representative of the plant community at the study site. The native mixture included 0.5 grams each of *Stipa pulchra* Hitchc. (bunch grass), *Artemisia californica* Less. (shrub), *Acmispon glaber* (Vogel) Brouillet (shrub), and *Eriogonum fasciculatum* Benth. (shrub). The non-native mixture included 0.5 grams each of *Avena fatua* Schreb. (grass), *Bromus hordeaceus* L. (grass), *Foeniculum vulgare* Mill. (forb), and *Brassica nigra* W.D.J.Koch (forb). We oven-dried seed in paper bags at 90°C for two weeks before measuring and deploying to prevent germination in the field and to remove moisture. After 48 hours in the field, we collected the seed remaining in the dishes and dried them at 45°C for two weeks to remove moisture. We then weighed seed again to find the mass of seed removed for each of the native and non-native mixtures.

We deployed each seed station for two consecutive days and nights. Limitation in field camera equipment meant that we could not deploy all seed stations simultaneously; instead, we monitored two stations at one time. With 28 seed stations, we were able to record 24-hour video surveillance of all stations over the course of one month. We repeated the study during three seasons: spring of 2010 (late May to mid-June); fall of 2010 (late October to mid-December); and winter of 2011 (mid-January to mid-February).

The Institutional Animal Care and Use Committee (Division of Research Affairs, Office of Graduate and Research Affairs, San Diego State University) approved of this research.

### Video Observation

**Fig 1:**
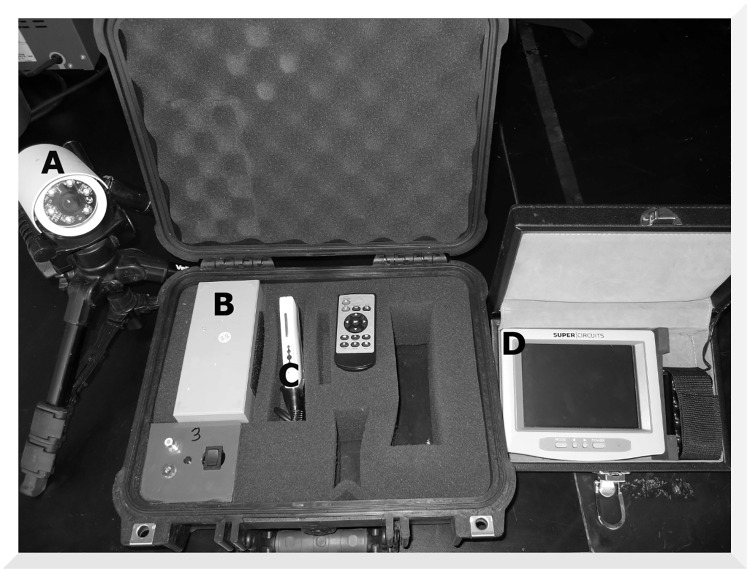
Digital camera system. The surveillance camera (A) is connected to a padded Pelican^™^ case, which contains a battery supply (B) and a mini-DVR (C). The monitor (D) can be connected to the system during setup in the field to ensure the seed station is properly focused within the camera’s field of view.

We designed a custom-built video recording system, which contains a surveillance camera (Standard Rugged Infrared LED Color Camera, Supercircuits, Inc., Austin, Texas) connected to a mini-DVR (AKR-100, Seorim Technology, South Korea) and either a lead-acid or lithium-ion battery (Fig 1). On a full charge, the batteries will allow continuous or motion-sensor video to be taken for about 24 hours at a time. Although the system had motion sensor capabilities, this setting often led to data storage failures. As a result, we employed the continuous-recording setting. We replaced batteries after each 24-hour recording session. Video footage could be screened at fairly high speeds (8x to 16x) using the AKR Player software without missing an animal visitation. It took three to six hours to review and record data for a typical 48-hour trial.

**Fig 2:**
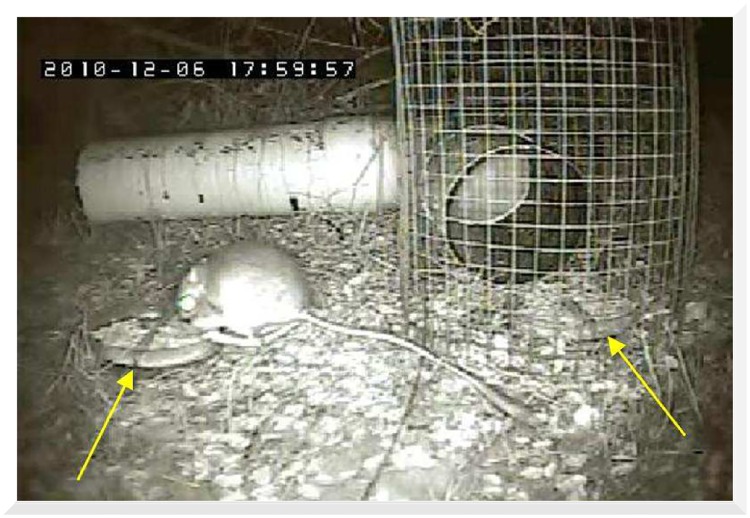
Seed dish visitation. A nighttime picture of a visit by *Dipodomys* sp. to a seed dish. The arrow indicates the location of the partition, which separates the native and non-native seed mixtures. The rodent is visiting the open dish, and an enclosed dish is visible inside the wire mesh cage. The PVC tube, bent at a 90-degree angle, is the only entrance to the enclosed seed dish. This is meant to prohibit entrance by rabbits and birds while permitting access to rodents.

We coupled each 48-hour seed predation trial with an *in situ* camera system so that every seed predation event was recorded (Fig 2). We situated the cameras approximately 40 cm from the seed stations, and positioned them such that the partition in the Petri dish faced the camera, making it possible to differentiate between native and non-native seed removal. We recorded the time the event began, the elapsed time of the event, the animal taxon (typically to the genus level), whether or not the animal visited a dish, the type of seed removed (native or non-native), and the type of dish visited (open or enclosed dish). The “open dish” refers to the dish exposed to all seed predators; the “enclosed dish” refers to the dish located inside the exclusion cage. We define a “detection” as an observation of an animal within the field of view of the camera; therefore, a detection did not necessarily mean the animal removed seed from a dish.

### Analysis

Before performing statistical analysis, we removed taxa that either visited seed dishes rarely or were not observed removing seed. For the seed removal part of the analysis, the mass of seed removed from each side of the dish was an experimental unit. Thus, there were four measurements per station: two seed types at each of two dish types. For the visitation and elapsed time analyses, each visit by a seed predator to a seed station was an experimental unit.

Seed removers exhibited different behaviors while removing seed from dishes. Certain genera were more likely to remove one seed and run away immediately (e.g., *Peromyscus*), while others would remain at the seed dish for minutes at a time removing multiple seeds (e.g., *Chaetidipus*). To account for this phenomenon, we evaluated seed and dish type preferences based on 1) the number of seed dish visits and 2) the amount of time a granivorous animal spent removing seed per visit (elapsed time). Finally, we combined seed removal measurements with video evidence of seed remover identities to determine whether the presence of certain genera for each 48-hour trial influenced the mass of seed removed in each seed/dish type.

We were able to discern removal from the native vs. non-native side of the seed dishes by strategic placement of the dishes (Fig 2). Animals may solely remove from the "native" or "non-native" side of the dish, or may remove from "both" sides during the same visit. Thus, seed type for analyses of video observations includes three levels (native, non-native, or both). Seed type for mass of seed removed compares only native vs. non-native seed removal. Dish type refers to open vs. enclosed dishes.

#### Video measurements: number of visits

To determine whether the number of visits varies by seed type, dish type, or genus, we used generalized linear mixed effects modeling with the lme4 package in R [21, 22] and the lsmeans package to perform pairwise comparisons [23]. The response variable was the number of visits (per season/station/genus/dish type/seed type combination), and the predictors are dish type, seed type, and genus. We used a Poisson distribution and a log-link function to account for the non-normal distribution of the response variable. The random intercept was season nested within station, accounting for temporal and spatial variability in dish visitation without sacrificing degrees of freedom. We removed June visitations from this analysis; summer had a very low number of visits compared to fall and winter, and the additional level of this random effect caused problems with model convergence.

We compared seven models based on additive and interactive effects between dish types, seed types, and genus presence: 1) the effect of genus only; 2) genus plus dish type; 3) genus plus seed type; 4) genus plus dish type plus seed type; 5) dish type plus the interaction between seed type and genus; 6) seed type plus the interaction between dish type and genus; and 7) interaction between seed type and genus plus the interaction between dish type and genus. We used Akaike information criterion (AIC) to select the strongest model, and least-squares means and contrasts to evaluate differences in the number of visits for seed types and genus of visitor.

#### Video measurements: elapsed time per visit

To evaluate elapsed time (time that visitors spend removing seed from dishes), we constructed linear mixed models with the nlme package in R [24, 22]. We used the log-transformed elapsed time as the response variable, with the fixed effects of genus, seed type, and dish type. We used the random intercept of station only (season nested within station did not improve model fit). We removed visitation by *Sylvilagus* due to low sample sizes. As a result, the analysis evaluating time spent removing seed focuses on preference for dish and seed type among the three rodent genera: *Chaetodipus*, *Peromyscus*, and *Dipodomys*.

We constructed the same seven models as described for "Video measurements: number of visits" and used AIC to select the strongest model. We performed least-squares means and contrasts to evaluate differences in the number of visits for seed types and genus of visitor.

#### Mass of seed removal with video measurements

To evaluate seed removal using video predictors, we took a linear mixed effects approach using the nlme package in R [24, 22]. We used mass of seed removed (g) as the response variable, with presence of each genus, seed type, and dish type as the fixed effects. We used season nested within station as the random intercept term. Finally, we added variance structures to account for heteroscedasticity for *Sylvilagus* presence and season. We used least-squares means and contrasts to evaluate differences in seed removal between genera.

We compared ten models, which are listed and described in Table 1. Aside from the interactions between seed type, dish type, and genera, this analysis also includes applicable genus-genus interactions (it was not possible to include a genus-genus interaction for genera that were never observed at the same station). This allowed us to account for concomitant effects of seed removal by multiple genera removing seed during a trial.

**Table 1:** Model comparisons for linear mixed-effects model using mass of seed removed as the response variable. Each model incorporated the random effect of season nested within station, and a variance structure to account for heteroscedasticity for *Sylvilagus* presence and season. Delta AIC values indicate the difference between the highest performing model and each of the competing models.

Model number	Model description	Model structure	logLik	Delta AIC
**1**	**Full model: Interactions between genera and dish type, plus interactions between genera and seed type, plus interactions among genera**	Pesp*Seed + Pesp*Dish + Seed*Dish + Disp*Seed + Disp*Dish + Chsp*Seed + Chsp*Dish + Sysp*Seed + Sysp*Dish + Disp*Pesp + Disp*Chsp + Pesp*Chsp + Pesp*Sysp + Chsp*Sysp	**22.12**	**0**
2	Interaction between genera and seed type, plus interactions among genera	Dish + Disp*Seed + Pesp*Seed + Chsp*Seed + Sysp*Seed + Disp*Pesp + Disp*Chsp + Pesp*Chsp + Pesp*Sysp + Chsp*Sysp	5.481	23.270
3	Interactions between genera and seed type, plus interactions between genera and dish type	Disp*Seed + Disp*Dish + Pesp*Seed + Pesp*Dish + Chsp*Seed + Chsp*Dish + Sysp*Seed + Sysp*Dish	4.376	25.478
4	Interactions between genera and dish type, plus interactions among genera	Seed + Disp*Dish + Pesp*Dish + Chsp*Dish + Sysp*Dish + Disp*Pesp + Disp*Chsp + Pesp*Chsp + Pesp*Sysp + Chsp*Sysp	2.612	29.006
5	Seed type plus interactions among genera	Dish + Seed + Disp*Pesp + Disp*Chsp + Pesp*Chsp + Pesp*Sysp + Chsp*Sysp	-8.731	43.693
6	Interactions between genera and seed type	Dish + Disp*Seed + Pesp*Seed + Chsp*Seed + Sysp*Seed	-12.19	48.602
7	Seed type plus interactions between genera and dish type	Seed + Disp*Dish + Pesp*Dish + Chsp*Dish + Sysp*Dish	-15.09	54.411
8	Seed type plus genera presence	Dish + Seed + Pesp + Chsp + Disp + Sysp	-26.40	69.035
9	Null model	Dish	-43.54	93.314
10	Seed type effect	Dish + Seed	-43.54	95.314

R^2^ (marginal) of full model: 0.667

R^2^ (conditional) of full model: 0.881

*Interaction terms of models

## Results

Small mammal detections (where an animal is visible within the camera's field of vision) were highly variable across taxa. The most common genera detected were deer mice and white-footed mice (*Peromyscus*; 672 total detections), kangaroo rats (*Dipodomys*; 202 detections), pocket mice (*Chaetodipus*; 127 detections), and cottontail rabbits (*Sylvilagus*; 96 detections). Woodrats (*Neotoma*) were detected 32 times; this small number of detections (and even fewer seed removal events) warranted the removal of this genus from analysis. Rare detections included birds, ants, one California vole (*Microtus californicus*), one striped skunk (*Mephitis mephitis*), and one black-tailed jackrabbit (*Lepus californicus*), none of which appeared to remove seed from the seed stations. It was difficult to determine via video footage whether ants were removing seed from the stations. However, we did not measure significant seed removal for trials during which we observed ants crawling in and around the seed dishes. The results and discussion will therefore focus on seed removal by rodent genera (*Peromyscus*, *Chaetodipus*, and *Dipodomys*) and *Sylvilagus*.

### Video measurements

The *number* of seed visits and the *time elapsed* per seed visit were modeled separately to look for nuanced differences in preference between seed types and dish types among the genera of visitors. For both the models, the additive model that includes all fixed effects (seed type, dish type, and genus) performed best; thus, the results described are extracted from the additive models. None of the interactions between genus and seed type or genus and dish type were important in describing the number of visits or time elapsed per visit.

#### Non-native vs. native seed visitation

**Fig 3:**
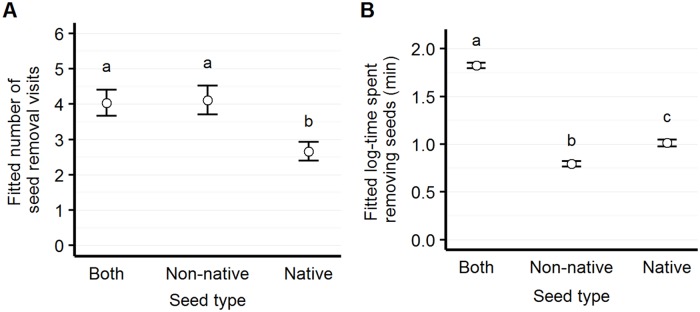
Number of visits and elapsed time by seed type. Model-fitted number of seed removal visits (panel A) and elapsed time per visit (panel B) for each of three possible seed "preference" scenarios: for each visit, the granivorous animal may visit "both" sides of a partitioned Petri dish; the "non-native" side only; or the "native" side only. Although animals remove non-native seeds more than native seeds, they spend more time per visit removing native than non-native seeds.

We recorded significantly more visits at both sides of the dish than for native seed only (Tukey pairwise comparison, z = 4.34, p<0.001), and more visits for non-native than native seed (Tukey pairwise comparison, z = 3.65, p<0.001). Similarly, we observed more time spent removing both seed types than either native or non-native seed (Tukey pairwise comparison, t = 4.99, p<0.001; t = 9.69, p<0.001, respectively); however, we found overall more time spent removing native than non-native seed (Tukey pairwise comparison, t = -3.26, p = 0.003) (Fig 3).

#### Open vs. enclosed dish visitation

**Fig 4:**
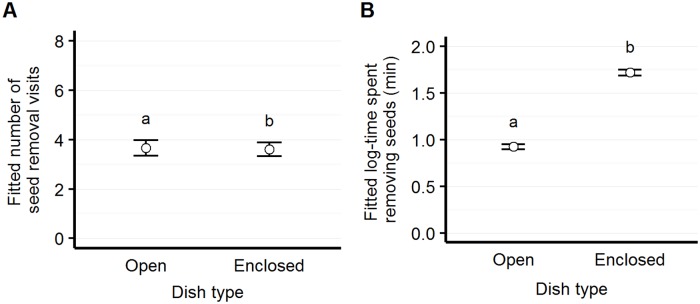
Number of visits and elapsed time by dish type. Model-fitted number of seed removal visits (panel A) and elapsed time per visit (panel B) for the two dish types: open (available to all seed predators); and enclosed (available only to rodents). Although animals remove seed more often in open dishes than enclosed dishes, they spend more time removing seed per visit at enclosed than open dishes.

We observed significantly more visits at open than enclosed dishes (z = -2.28, p = 0.022); *Sylvilagus* visited the open dish exclusively. However, we found that visitors spent more time removing from the enclosed than the open dishes (t = 8.76, p<0.001) (Fig 4).

#### Visitation by genus

**Fig 5:**
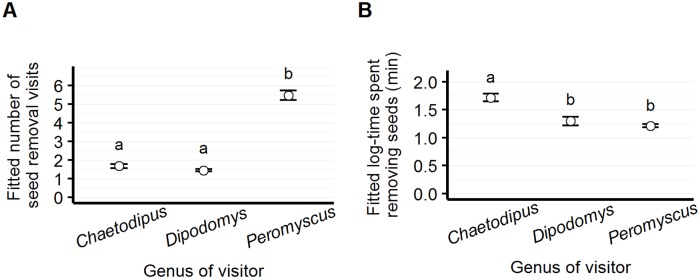
Number of visits and elapsed time by genus. Model-fitted number of seed removal visits (panel A) and elapsed time per visit (panel B) for three rodent genera (*Sylvilagus* was removed from this analysis due to sample size limitations). Although *Peromyscus* have a greater number of visits than *Chaetodipus* and *Dipodomys*, they spend less time removing seed per visit than *Chaetodipus*.

We found that the number of visits varied significantly by genus, where *Peromyscus* had more visits than *Chaetodipus* and *Dipodomys* (Tukey pairwise comparison, z = -6.77, p<0.001; z = -6.38, p<0.001, respectively). However, *Chaetodipus* spent significantly more time removing seed than *Peromyscus* (Tukey pairwise comparison, t = 4.74, p<0.001) (Fig 5).

### Mass of seed removed with video measurements

**Fig 6:**
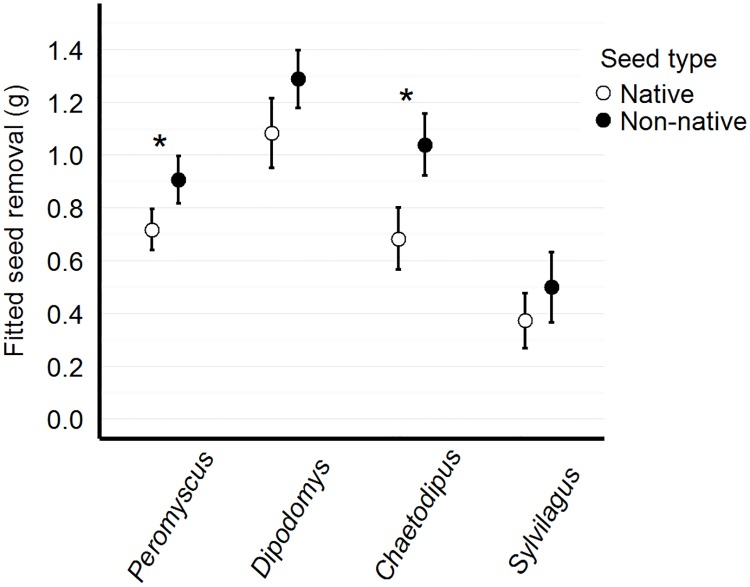
Mass of seed removal by genus and seed type. Model-fitted seed removal (in grams) for native and non-native seed mixtures based on the presence of certain genera of seed predators. Although all seed predators remove more non-native than native seed, only *Peromyscus* and *Chaetodipus* exhibit significant preference for the non-native seed mixture.

**Fig 7:**
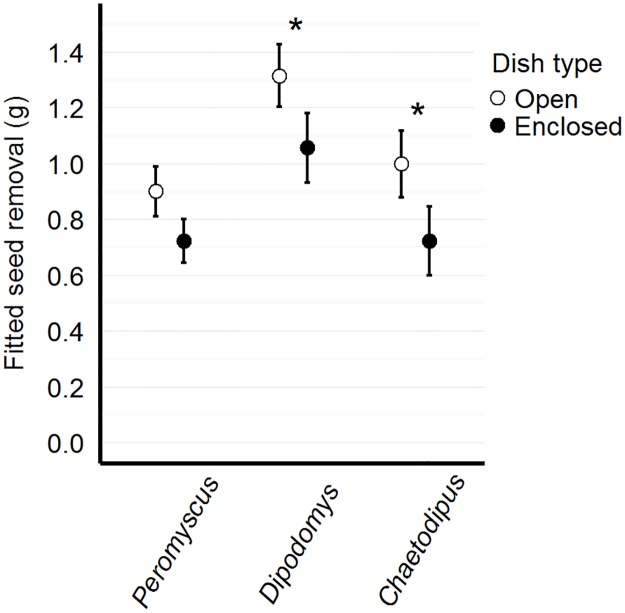
Mass of seed removal by genus and dish type. Model-fitted seed removal (in grams) for open and enclosed dish types based on the presence of certain genera of seed predators. Although all seed predators remove more seed from open dishes, only *Dipodomys* and *Chaetodipus* visit the open dish significantly more than the enclosed dish.

**Fig 8:**
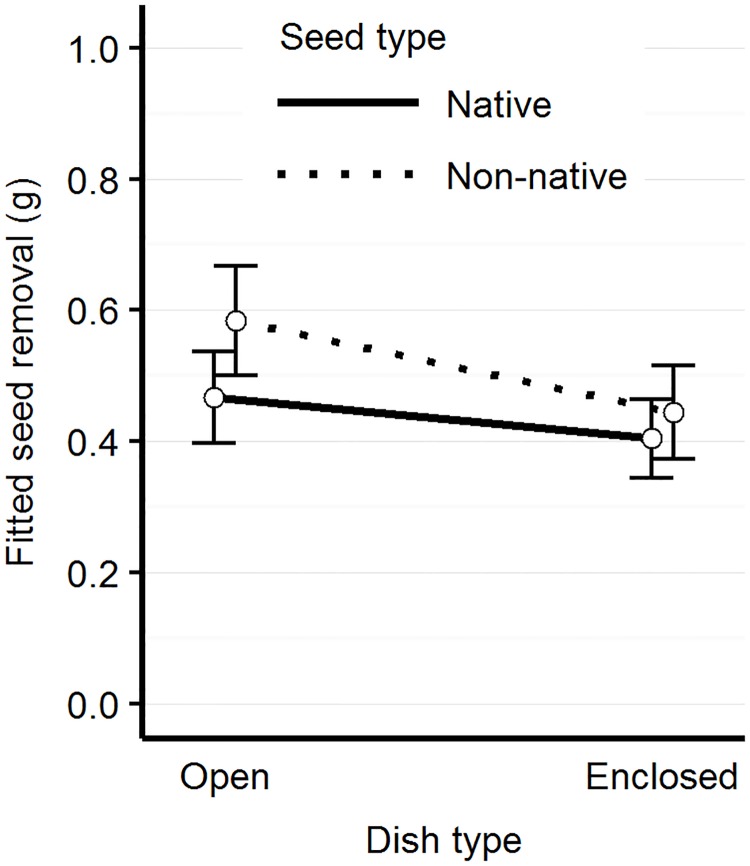
Mass of seed removal by seed and dish type. Model-fitted seed removal (in grams) for native and non-native seed mixtures for each dish type. We measured a greater preference for non-native seed in the open dish than in the enclosed dish.

The full model performed best (Table 1), incorporating all two-way interactions between genera and seed type, genera and dish type, seed type and dish type, and genus-genus interactions. We found genus-specific patterns of apparent seed and dish preference. When *Chaetodipus* and *Peromyscus* were present in a trial, significantly more non-native seed was removed (t = 4.28, p<0.001; t = 2.09, p = 0.039, respectively) (Fig 6). When *Dipodomys* and *Chaetodipus* are present, significantly more seed was removed from open than enclosed dishes (t = -2.49, p = 0.014; t = -2.55, p = 0.012, respectively) (Fig 7). We did not detect any interactions between *Peromyscus* presence and seed removal by dish type. We also found a significant interaction between seed and dish type (t = -2.45, p = 0.015), where more non-native seed is removed from the open than the enclosed dish (Tukey pairwise comparison, t ratio = 6.42, p<0.001) (Fig 8, Table 2).

**Table 2:** Results of the highest performing model, indicated in Table 1. Parameter estimates, their standard errors, and the p-values for each effect are included. P-values less than 0.05 for interaction effects are in bold.

Model effect	Estimate	p-value
Main effects		
Genera (reference		
level: Absent		
Pesp	0.277 ± 0.121	0.0353
Disp	1.316 ± 0.195	<0.001
Chsp	0.552 ± 0.183	0.0083
Sysp	0.150 ± 0.153	0.341
Dish type (reference	0.0524 ± 0.0244	0.0329
level: Open dish)		
Seed type (reference	-0.0003 ± 0.0244	0.990
level: Native seed)		
Interactions		
**Pesp: Seed type**	**0.0805 ± 0.0386**	**0.0386**
**Chsp: Seed type**	**0.322 ± 0.0752**	**<0.001**
Disp: Seed type	0.153 ± 0.0875	0.0829
Sysp: Seed type	0.0494 ± 0.0590	0.404
Pesp: Dish type	-0.0491 ± 0.0386	0.205
**Chsp: Dish type**	**-0.192 ± 0.0752**	**0.0118**
**Disp: Dish type**	**-0.218 ± 0.0875**	**0.0138**
**Sysp: Dish type**	**-0.232 ± 0.0590**	**<0.001**
**Seed type: Dish type**	**-0.0769 ± 0.0314**	**0.0154**
Pesp: Disp	-0.431 ± 0.258	0.115
Pesp: Chsp	0.161 ± 0.232	0.497
Pesp: Sysp	0.459 ± 0.273	0.113
**Chsp: Disp**	**-1.808 ± 0.369**	**<0.001**
Chsp: Sysp	-0.543 ± 0.283	0.0733

## Discussion

By performing a study of selective seed predation while recording all seed removal with digital cameras, we found that the animals removing seed from the enclosed dish were a subset of the community we expected would use the exclusion equipment. We documented "tube-avoidance” behavior by rodents in terms of the number of visits to open vs. enclosed dishes, as well as the mass of seed removed in open vs. enclosed dishes when rodent taxa were present. Given the prevalence of using exclusion equipment for inferring patterns of seed predation without using video observation (e.g., [12–14]), our findings imply that results from such studies may not be interpreted accurately.

Although seed predators were more likely to visit the non-native than the native side of the dish, overall they spend more *time* per visit removing seed from the native side. It is unclear why this pattern emerged. Another study found that rodents are more likely to consume soft-shelled than hard-shelled seed; the latter were instead cached in hoards [25]. Similarly, Xiao et al. [26] found that larger seed were more likely than smaller seeds to be hoarded. Rodents may be using some kind of criteria (e.g., shell hardness or seed size) to determine whether to consume or cache a seed. If they prefer to eat native seed on-site, while caching the larger non-native seed, this may explain differences in elapsed time between native and non-native removal. Rodents with cheek pouches can quickly retrieve a relatively large number of seeds in one visit for later caching. Alternatively, native seed may take longer to husk than the larger non-native seed. If this were the case, it would explain 1) longer elapsed time spent removing native seed and 2) preference for non-native seed by certain genera, since optimal foraging theory predicts that seed predators minimize the amount of energy spent processing food resources [27].

Similarly, there were a greater number of visits to the open dish, but seed predators spent more time removing seed per visit at the enclosed dish. If this result was simply reflective of the subset of rodents removing seed from the enclosed dish, we would expect shorter visits in the enclosed dish–*Peromyscus* spent less time at dishes per visit than *Chaetodipus*, and were also more likely to use the enclosed dish. One possibility is that the proximity of the tube as an escape from predators meant that those removing seed were able to spend more time foraging [28]. Others have found that when confronted with scents mimicking predators, rodents foraged less efficiently [29]. This implies that perceived safety from predators may alter foraging behavior.

In this study, the open dishes had a greater overall mass of seed removed, as well as a greater removal of non-native seed. The interpretation of these results, without video observation, would lead to the conclusion that *Sylvilagus* spp. (too large to enter rodent-only exclosures) were important seed predators during the fall and winter months, and exhibited preference for non-native seed. However, we saw very few *Sylvilagus* visits to seed stations during the fall and winter sampling period, and no evidence of *Sylvilagus* preference for non-native seed. Our interpretation is that the combined efforts of *Dipodomys* and *Chaetodipus* (by being more likely to visit open than enclosed dishes) and *Sylvilagus* (by only visiting the open dishes) inflate the mass of seed removed from open dishes. Furthermore, *Chaetodipus*–not *Sylvilagus*–exhibited preference for non-native seed, which may have accounted for the greater removal of non-native seed from open dishes.

Many seed removal studies attempt to partition seed removal between bird, rodent, and insect granivores (e.g., [7, 14]). Fewer studies attempt to isolate removal patterns between smaller taxonomic units (e.g., between rodent genera). When using video observation, studies may determine which rodent genera are participating in seed removal, providing a qualitative supplement to measures of seed removal (e.g., [30]). But unless cameras are monitoring all experimental units, it is difficult to assign different removal patterns among genera. Those that are able to differentiate seed removal among rodent genera are able to do so because the genera have different body sizes or daily activity patterns. Researchers can equip exclosure cages with holes of different sizes, where for example only small-bodied small mammals can access a particular dish [13]. Researchers can also check and measure seed dishes twice daily to account for diurnal vs. nocturnal seed removal patterns [10, 11]. However, the persistent problem is that if rodent genera with similar body sizes and daily activity patterns exhibit different seed removal behaviors, this remains unseen in studies adopting indirect inference. Differentiating between similar genera provides a more nuanced approach and may yield important insights when these smaller taxonomic units exhibit different patterns of seed removal.

We observed genus-specific differences in seed type preference among crepuscular and nocturnal visitors, with non-native seed preference exhibited by *Peromyscus* and *Chaetodipus*, but not *Sylvilagus* or *Dipodomys*. One implication of such a result is that in seed-limited systems, population fluctuations of certain rodent genera may influence aboveground plant community dynamics. For the current study system, the implication is that *Peromyscus* and *Chaetodipus* may have undue influence on the invasion of non-native plants. However, genus-specific selectivity of seed may vary by system; indeed, after excluding a guild of *Dipodomys* species, Brown and Heske [3] measured significant increase in the cover of annual grasses in a shrub-dominated system. In their system, *Dipodomys* may have selectively predated the larger annual grass seed, thereby inhibiting their germination and proliferation.

Seed removers exhibited differences in seed and dish type selectivity: only *Peromyscus* and *Chaetodipus* preferred non-native seed; and of the taxa that used the enclosed dish, *Peromyscus* was the only genus that did not exhibit tube avoidance. The implication is that without the benefit of video evidence, the seed removal measured from the enclosed dish would be interpreted as the measure of seed selectivity by the full rodent community. In this study, a subset of the rodent community did not visit the enclosed dish at the same frequency or remove as much seed mass from the enclosed dish. *Peromyscus*, by freely visiting the enclosed dish, may be driving seed removal patterns from the enclosed dish, and the rest of the seed removers may be driving patterns observed from the open dish. Non-native seed selectivity measured from the enclosed dish was weaker than that of the open dish, which may mean that *Chaetodipus–*by avoiding the enclosed dish and preferring non-native seed–was driving seed selectivity in the open dish.

It is unclear what aspect of the field equipment induced this unanticipated bias in attendance at the seed stations. It is possible that the PVC tube itself influenced the apparent avoidance by certain rodent taxa, and this occurrence may be eradicated by adopting another exclusion approach. For example, other studies of seed predation attempting to isolate removal by different rodent taxa cut holes of varying sizes in wire mesh (or plastic container) exclosures (e.g., [13, 31]). Still others will use wire mesh of different sizes, with larger mesh being used to allow a wider community of granivorous animals to participate in seed removal [17, 32]. Connolly et al. [7] excluded bird from rodent seed removal by adding a wire “canopy” four centimeters above the seed dish. Additionally, the proximity of the two dishes (about 40cm) meant that the easy visibility and accessibility of the open dish increased participation in lieu of the more challenging or unnoticed entrance to the enclosed dish. If the dishes were spaced farther apart, it is possible the enclosed dish would have higher participation when a more visible alternative is not available. It would be useful to pair these alternative approaches with video observation to determine whether or not they induce a visitor bias.

Given that the current study used two-day trials, rodents may not have had ample time to grow accustomed to the equipment, leading to preferential removal from the open dish. This effect may have lessened with a longer trial period or a period of habituation before the trial began. Lobo et al. [31] installed their seed predation equipment in the field three days prior to the trial to habituate seed predators. The equipment used in the current study had remained at the field site since Braswell's [14] study, so theoretically the animals living nearby would have been accustomed to its presence. However, without seed addition, seed predators likely had little reason to use the PVC tubes to gain access inside the cages until the start of the trials.

Although many other studies in North America show rodents to be the primary granivores [15, 16], it should be noted that birds may take longer to find seed depots than small mammals, thus their influence on seed predation may be underestimated during short-term studies [33]. It follows that the importance of bird granivory may have been underestimated in this study. With a limited trial period of two days, birds may not have had ample time to identify the stations as a food resource. Others have found that birds do not often show interest in seed offered in Petri dishes, and that using larger seed depots may be more appropriate [30].

Ecologists are increasingly using video observation of seed predation, where studies supplement indirect observation with video observations of behavior for at least a subset of the experimental units (e.g., [30, 34, 35]). The results of this study further illustrate the value of video observation for studies of seed predation: this approach 1) provided a means to evaluate assumptions about the effects of *in situ* equipment on the behaviors of granivorous animals; and 2) allowed us to tease apart patterns of seed predation among smaller taxonomic units (rodent genera) than indirect approaches. In the case of seed predation patterns, it is difficult to interpret seed removal without the benefit of video observation, particularly when the protocol involves exclusion equipment that the target animal community may avoid using.
